# Mitochondrial Genome Sequence of the Galápagos Endemic Land Snail *Naesiotus nux*

**DOI:** 10.1128/genomeA.01362-15

**Published:** 2016-01-21

**Authors:** Samuel S. Hunter, Matthew L. Settles, Daniel D. New, Christine E. Parent, Alida T. Gerritsen

**Affiliations:** a Institute for Bioinformatics and Evolutionary Studies (IBEST), University of Idaho, Moscow, Idaho, USA; b Department of Biological Sciences, University of Idaho, Moscow, Idaho, USA

## Abstract

We report herein the draft mitochondrial genome sequence of *Naesiotus nux*, a Galápagos endemic land snail species of the genus *Naesiotus*. The circular genome is 15 kb and encodes 13 protein-coding genes, 2 rRNA genes, and 21 tRNA genes.

## GENOME ANNOUNCEMENT

Land snails are known for their remarkable diversity and high levels of endemism on island systems (1). In Galápagos, the land snail fauna comprises 103 endemic species distributed in 13 genera (2). More than 70 of these species belong to the genus *Naesiotus* (family *Bulimulidae*), forming the most species-rich adaptive radiation of these islands (3). *Naesiotus nux*, the type species of the genus, was first described by W. J. Broderip in 1832 based on specimens collected on Floreana Island by Hugh Cuming in 1830 (4). *Naesiotus* land snails have successfully colonized all major islands and several small islets on Galápagos, and populations can be found in all land habitats, except for sandy beaches and barren lava flows (5). Although some species were originally described as having multi-island distributions, recent field and genetic work suggests that most Galápagos *Naesiotus* spp. are single-island endemic (5). This suggests that current species diversity in the Galápagos group is likely to be underestimated and makes current genomics studies critical in assessing conservation strategies.

Total genomic DNA was isolated from a sample of tissue preserved in dimethyl sulfoxide from a specimen of *N. nux* from San Cristobal Island, Galápagos, with the Qiagen DNeasy blood and tissue kit. Two individually bar-coded genomic libraries were prepared on the Apollo 324 platform with differing insert sizes (700 to 800 bp and 800 to 1,000 bp) through mechanical shearing (Covaris M-220) and gel extraction. Reads were sequenced on an Illumina MiSeq sequencer with 2 × 300-bp reads at the University of Idaho’s IBEST Genomics Resources Core. Shotgun sequencing of the genomic libraries resulted in 87× coverage of the genome.

Following sequencing, reads were cleaned using a custom bioinformatics pipeline to remove duplicate reads, trim off low-quality bases and sequence adapters from read ends, and overlap pairs using FLASH (6). The mitochondrial genome was then assembled using the ARC software package (https://github.com/ibest/ARC), which uses reference-seeded, iterative assemblies. The *Ascobulla fragilis* mitochondrial genome (NC012428) was used to initially seed the ARC assembly.

Following assembly, NCBI BLAST was used to identify a set of related mitochondrial sequences from *Biomphalaria glabrata* (AY380531), *Thuridilla gracilis* (DQ991939), *Biomphalaria tenagophila* (EF433576), *Succinea putris* (JN627206), *Ascobulla fragilis* (NC012428), and *Albinaria coerulea* (X83390). These sequences were linearized and oriented to all start with 16S rRNA and then aligned with MAFFT version 7.017 (7). Annotations were predicted from the MITOS web server (8); annotations and open reading frames were later refined and confirmed by constructing a custom BLAST database in Geneious version 7.0 (9) (http://www.geneious.com) from the 6 reference species.

The mitochondrial DNA (mtDNA) genome of *N. nux* is a circular DNA molecule of 15,197 bp. The G+C content is 26.7%. Predicted annotations from both Geneious and MITOS include common respiratory genes (*atp6*, *atp8*, *cob*, *cox1*, *cox2*, *cox3*, *nad1*, *nad2*, *nad3*, *nad4*, *nad4l*, *nad5*, and *nad6*), 2 rRNA genes (large and small subunits), and 21 tRNA genes. Alignments to other gastropod genomes range in percent identity from 52.136% to 84.316%, and phylogenetic analysis in Geneious indicates that *N. nux* is most closely related to *Albinaria coerulea*.

### Nucleotide sequence accession number.

The mtDNA genome sequence has been deposited in GenBank under the accession number KT821554.
